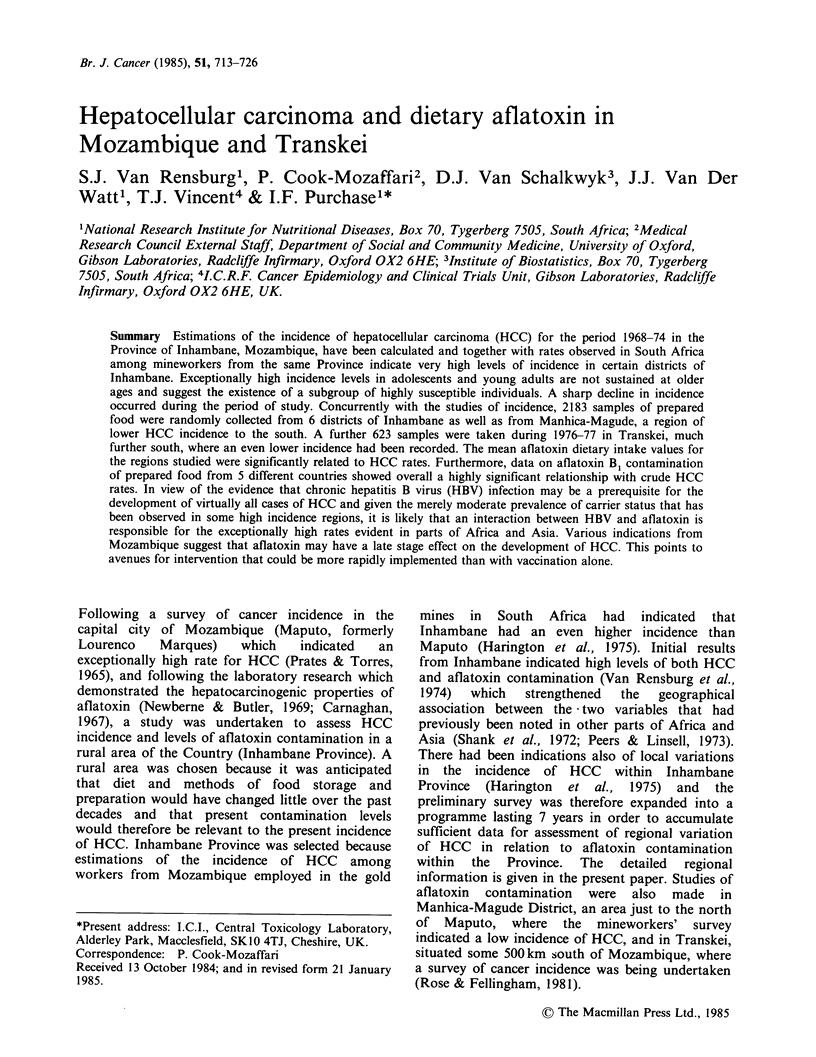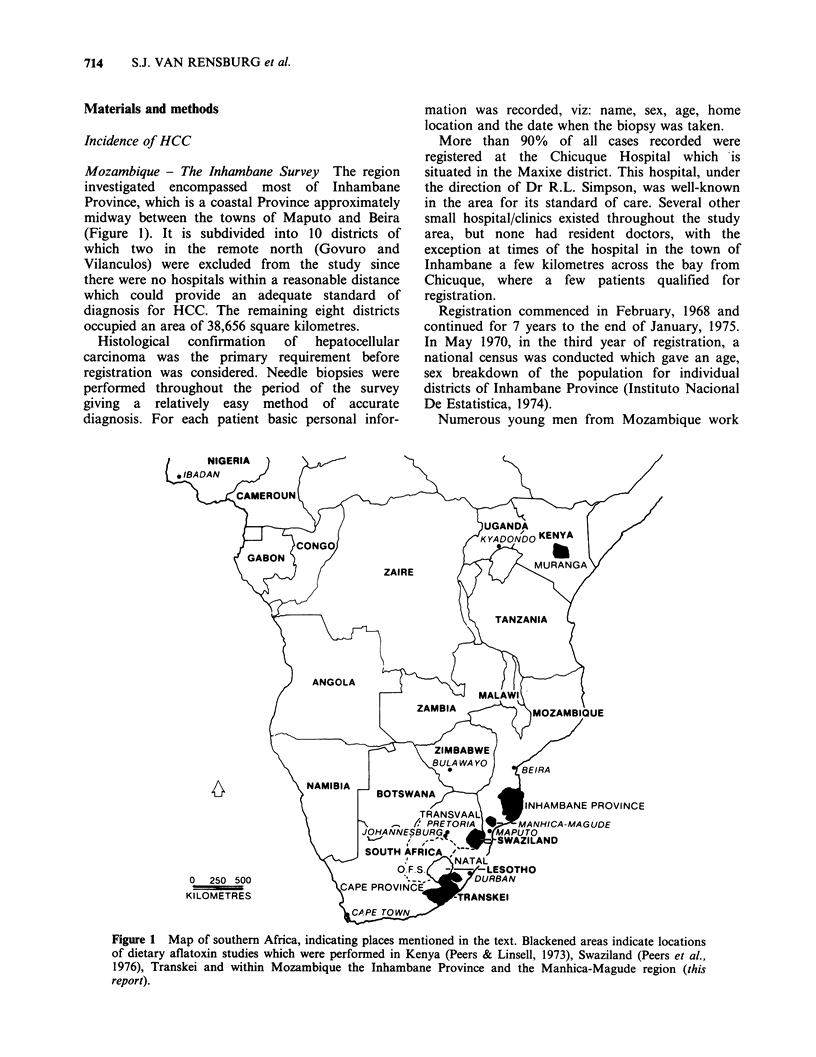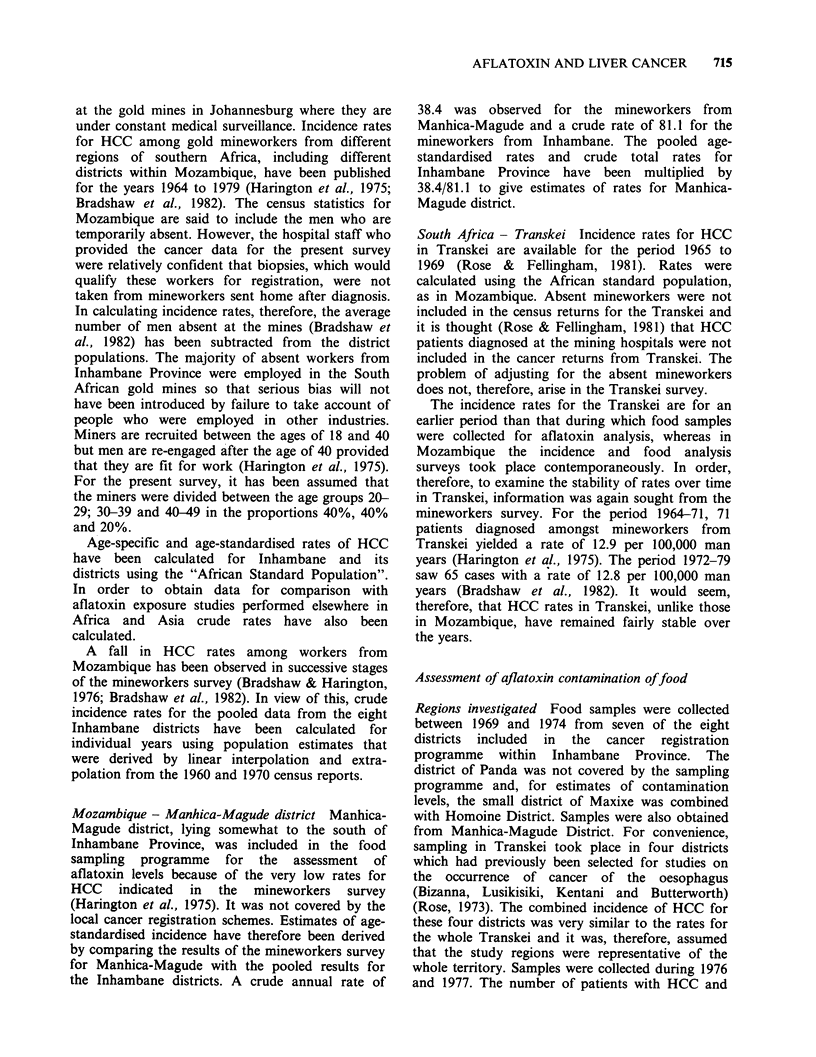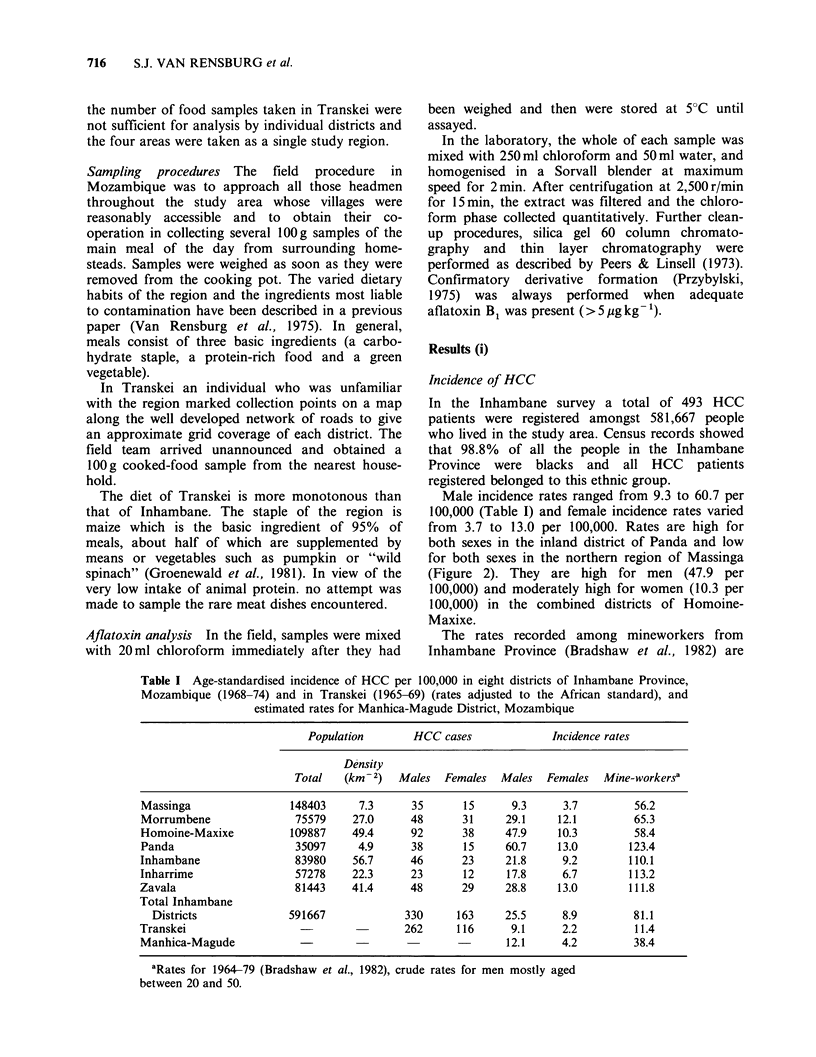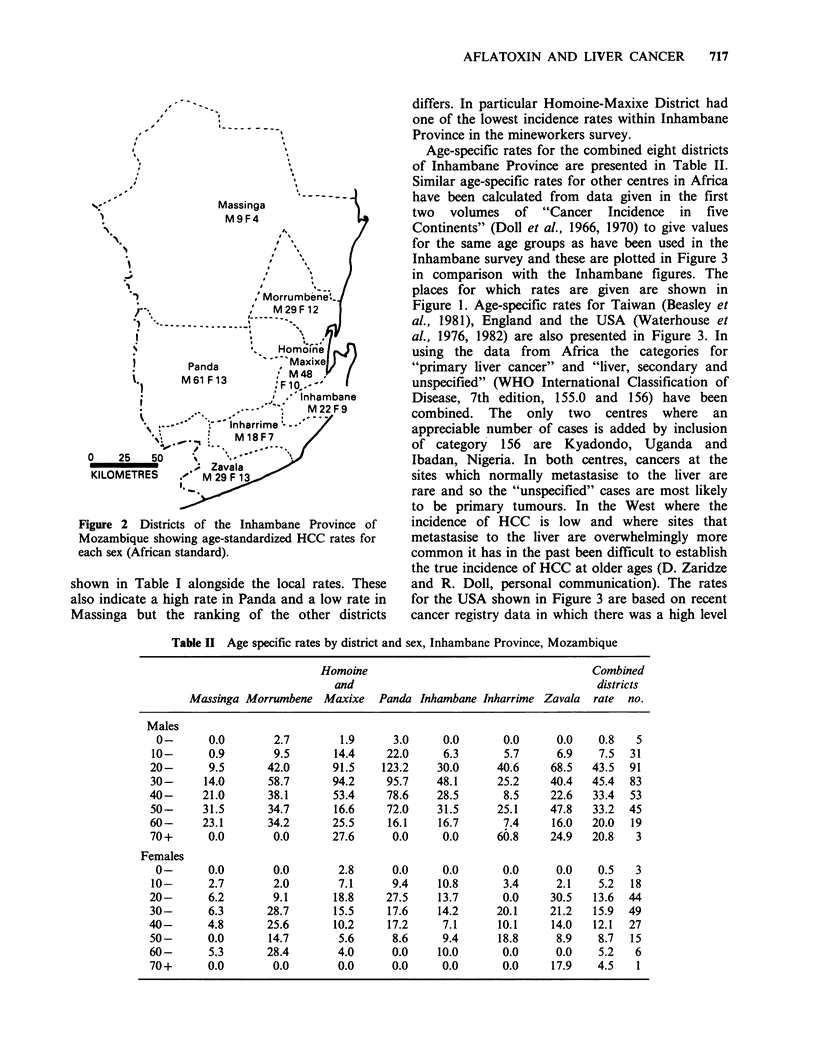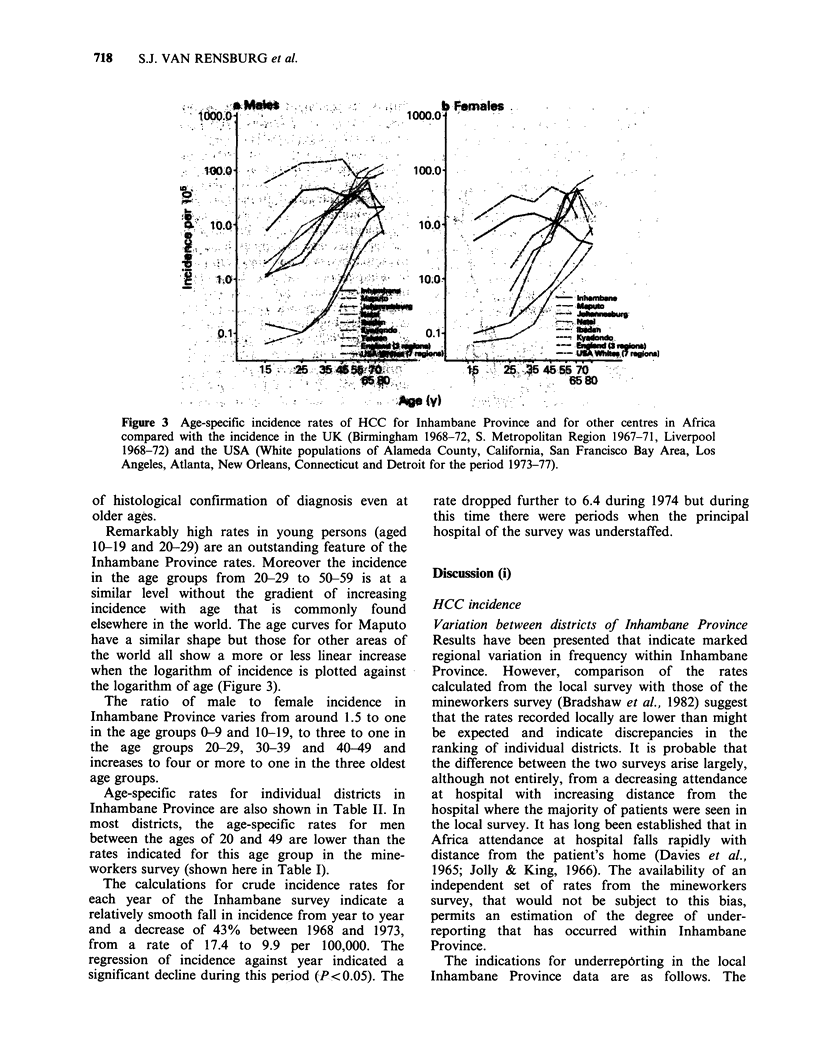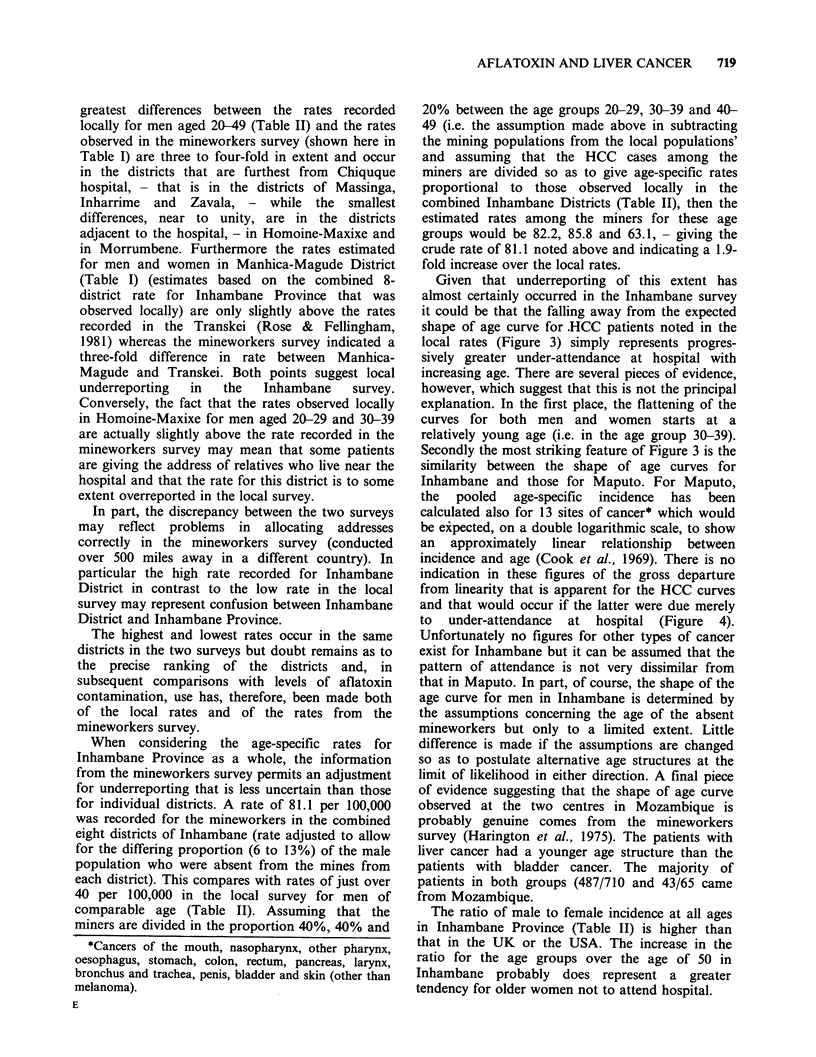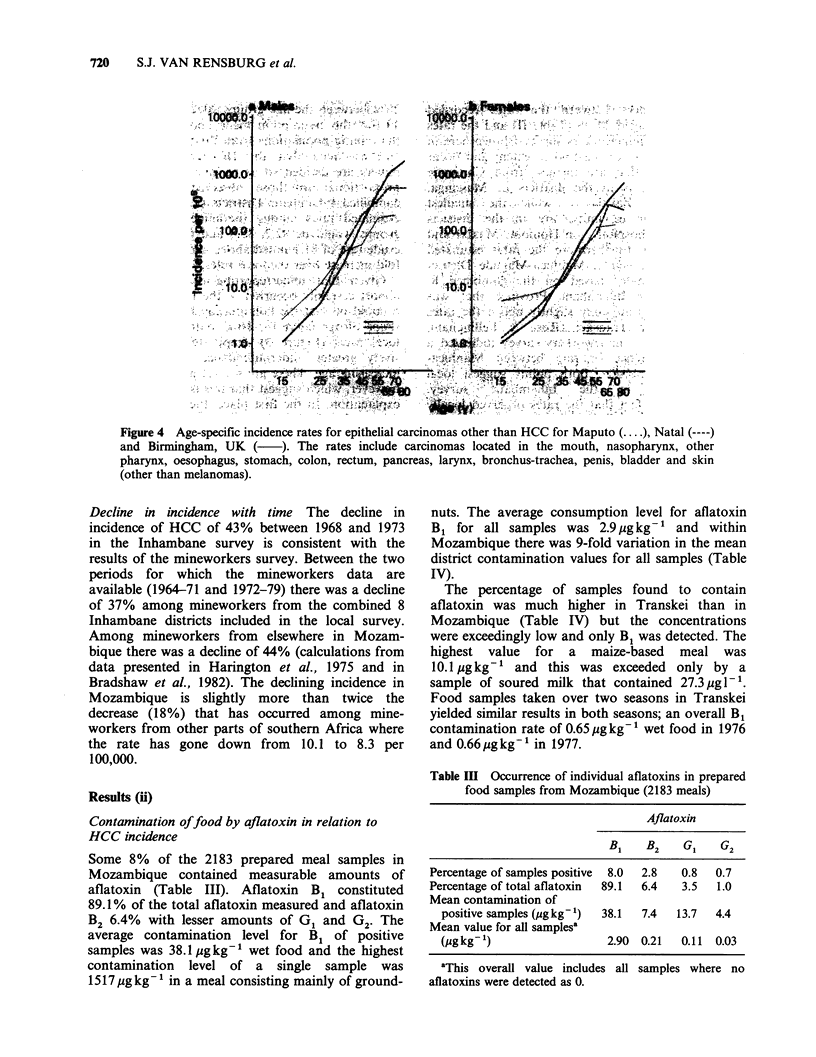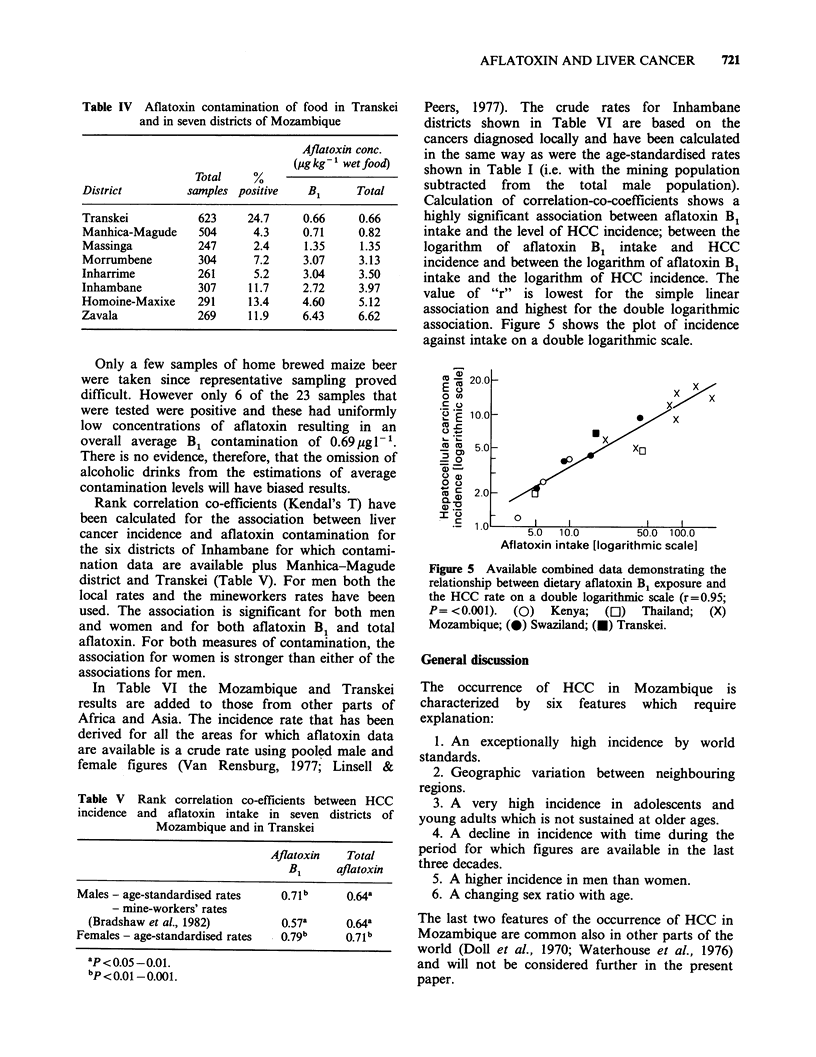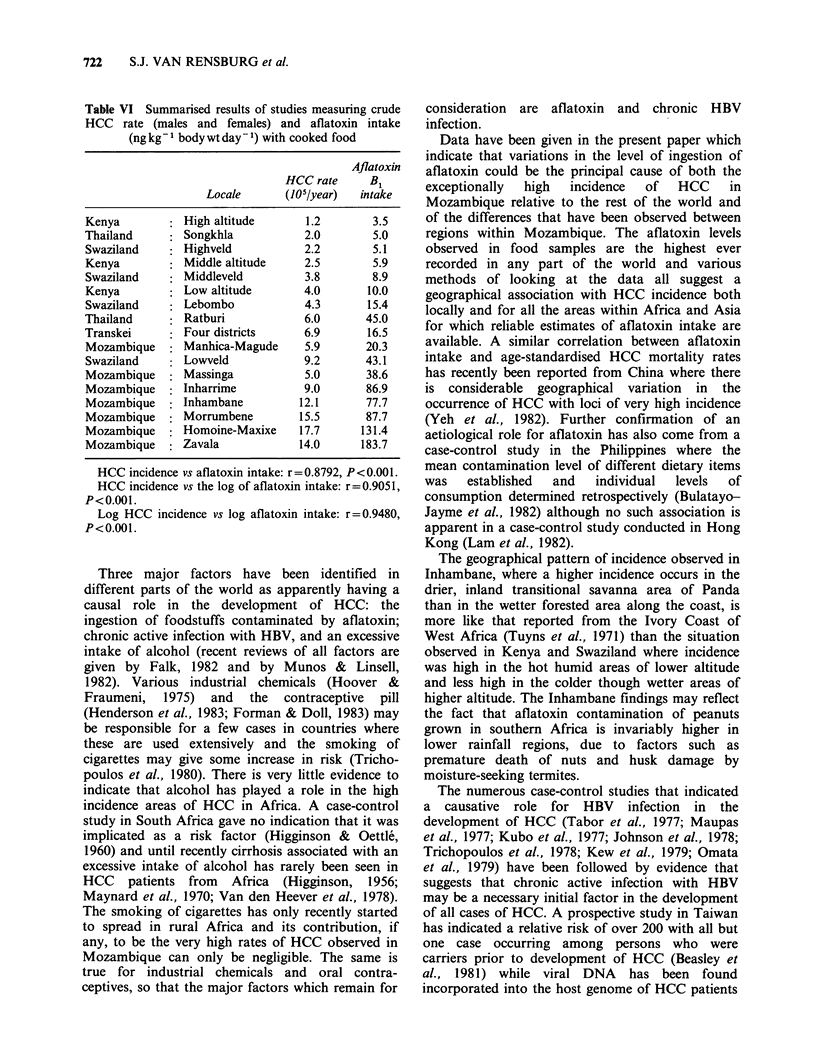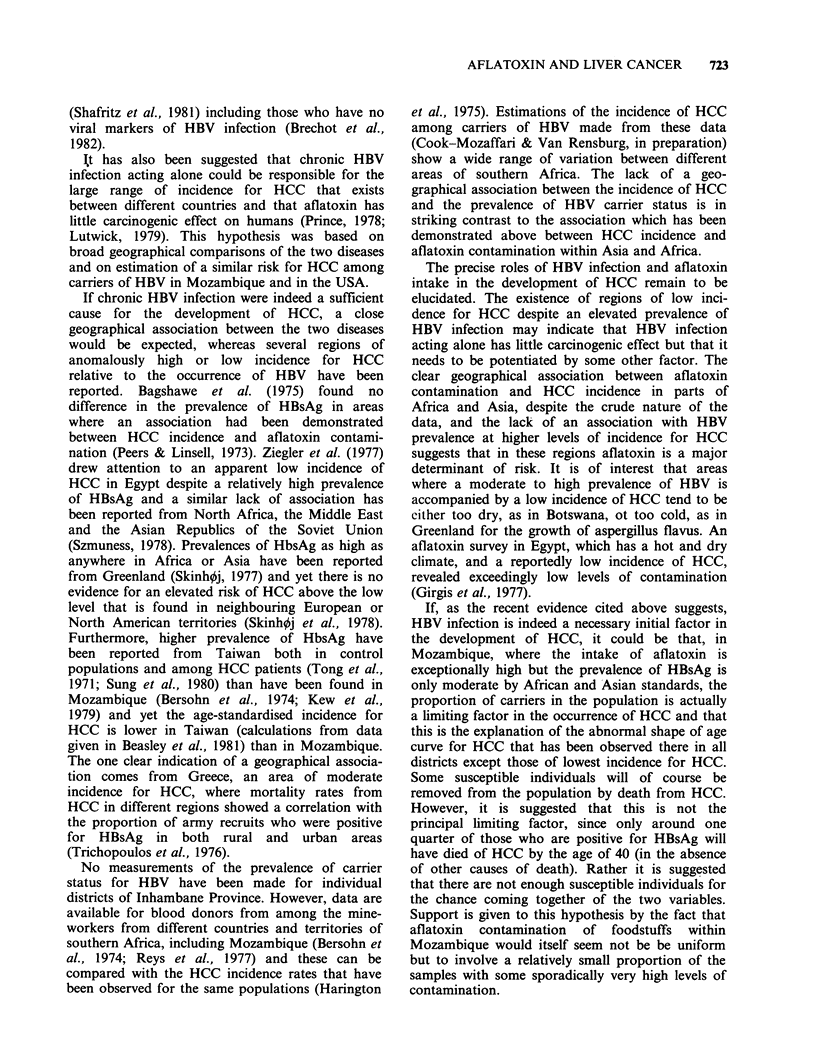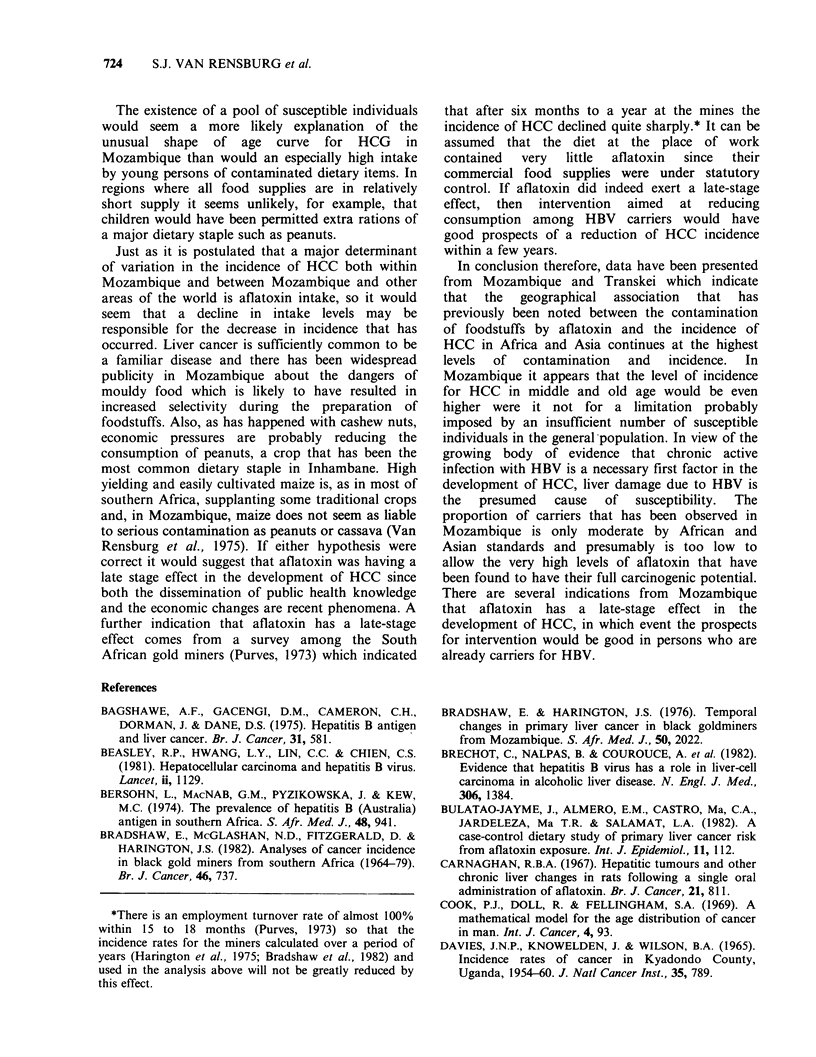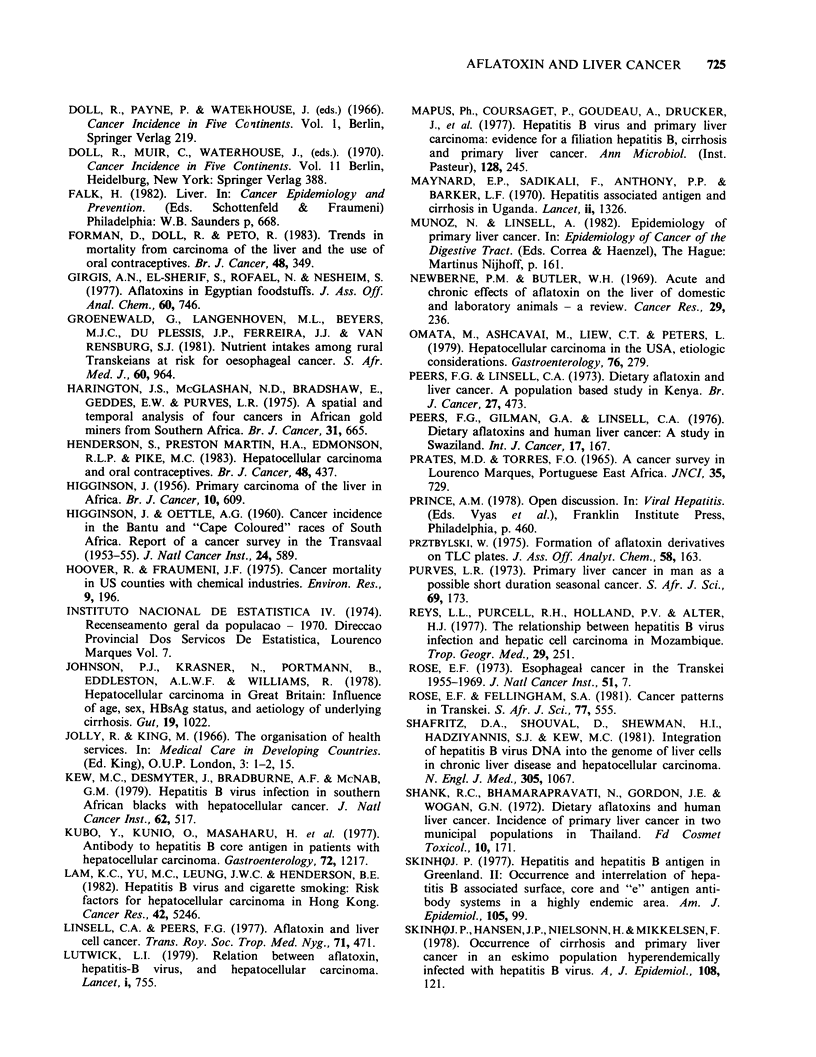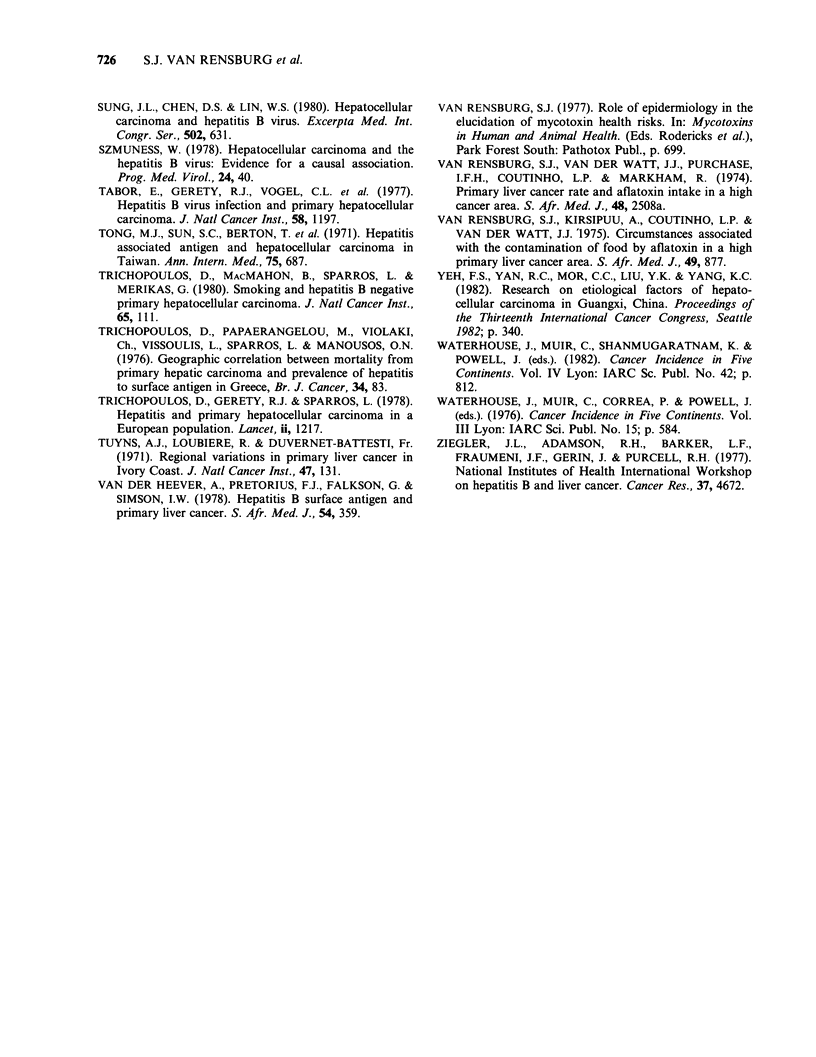# Hepatocellular carcinoma and dietary aflatoxin in Mozambique and Transkei.

**DOI:** 10.1038/bjc.1985.107

**Published:** 1985-05

**Authors:** S. J. Van Rensburg, P. Cook-Mozaffari, D. J. Van Schalkwyk, J. J. Van der Watt, T. J. Vincent, I. F. Purchase

## Abstract

Estimations of the incidence of hepatocellular carcinoma (HCC) for the period 1968-74 in the Province of Inhambane, Mozambique, have been calculated and together with rates observed in South Africa among mineworkers from the same Province indicate very high levels of incidence in certain districts of Inhambane. Exceptionally high incidence levels in adolescents and young adults are not sustained at older ages and suggest the existence of a subgroup of highly susceptible individuals. A sharp decline in incidence occurred during the period of study. Concurrently with the studies of incidence, 2183 samples of prepared food were randomly collected from 6 districts of Inhambane as well as from Manhica-Magude, a region of lower HCC incidence to the south. A further 623 samples were taken during 1976-77 in Transkei, much further south, where an even lower incidence had been recorded. The mean aflatoxin dietary intake values for the regions studied were significantly related to HCC rates. Furthermore, data on aflatoxin B1 contamination of prepared food from 5 different countries showed overall a highly significant relationship with crude HCC rates. In view of the evidence that chronic hepatitis B virus (HBV) infection may be a prerequisite for the development of virtually all cases of HCC and given the merely moderate prevalence of carrier status that has been observed in some high incidence regions, it is likely that an interaction between HBV and aflatoxin is responsible for the exceptionally high rates evident in parts of Africa and Asia. Various indications from Mozambique suggest that aflatoxin may have a late stage effect on the development of HCC. This points to avenues for intervention that could be more rapidly implemented than with vaccination alone.


*Br. J. Cancer* (1985), **51**, 713–726

### Hepatocellular carcinoma and dietary aflatoxin in Mozambique and Transkei

S.J. Van Rensburg[1], P. Cook-Mozaffari[2], D.J. Van Schalkwyk[3], J.J. Van Der Watt[1], T.J. Vincent[4] & I.F. Purchase[1]*

[1]*National Research Institute for Nutritional Diseases, Box 70, Tygerberg 7505, South Africa*; [2]*Medical Research Council External Staff, Department of Social and Community Medicine, University of Oxford, Gibson Laboratories, Radcliffe Infirmary, Oxford OX2 6HE*; [3]*Institute of Biostatistics, Box 70, Tygerberg 7505, South Africa*; [4]*I.C.R.F. Cancer Epidemiology and Clinical Trials Unit, Gibson Laboratories, Radcliffe Infirmary, Oxford OX2 6HE, UK.*

**Summary** Estimations of the incidence of hepatocellular carcinoma (HCC) for the period 1968–74 in the Province of Inhambane, Mozambique, have been calculated and together with rates observed in South Africa among mineworkers from the same Province indicate very high levels of incidence in certain districts of Inhambane. Exceptionally high incidence levels in adolescents and young adults are not sustained at older ages and suggest the existence of a subgroup of highly susceptible individuals. A sharp decline in incidence occurred during the period of study. Concurrently with the studies of incidence, 2183 samples of prepared food were randomly collected from 6 districts of Inhambane as well as from Manhica-Magude, a region of lower HCC incidence to the south. A further 623 samples were taken during 1976–77 in Transkei, much further south, where an even lower incidence had been recorded. The mean aflatoxin dietary intake values for the regions studied were significantly related to HCC rates. Furthermore, data on aflatoxin $B_1$ contamination of prepared food from 5 different countries showed overall a highly significant relationship with crude HCC rates. In view of the evidence that chronic hepatitis B virus (HBV) infection may be a prerequisite for the development of virtually all cases of HCC and given the merely moderate prevalence of carrier status that has been observed in some high incidence regions, it is likely that an interaction between HBV and aflatoxin is responsible for the exceptionally high rates evident in parts of Africa and Asia. Various indications from Mozambique suggest that aflatoxin may have a late stage effect on the development of HCC. This points to avenues for intervention that could be more rapidly implemented than with vaccination alone.

Following a survey of cancer incidence in the capital city of Mozambique (Maputo, formerly Lourenco Marques) which indicated an exceptionally high rate for HCC (Prates & Torres, 1965), and following the laboratory research which demonstrated the hepatocarcinogenic properties of aflatoxin (Newberne & Butler, 1969; Carnaghan, 1967), a study was undertaken to assess HCC incidence and levels of aflatoxin contamination in a rural area of the Country (Inhambane Province). A rural area was chosen because it was anticipated that diet and methods of food storage and preparation would have changed little over the past decades and that present contamination levels would therefore be relevant to the present incidence of HCC. Inhambane Province was selected because estimations of the incidence of HCC among workers from Mozambique employed in the gold mines in South Africa had indicated that Inhambane had an even higher incidence than Maputo (Harington *et al.*, 1975). Initial results from Inhambane indicated high levels of both HCC and aflatoxin contamination (Van Rensburg *et al.*, 1974) which strengthened the geographical association between the two variables that had previously been noted in other parts of Africa and Asia (Shank *et al.*, 1972; Peers & Linsell, 1973). There had been indications also of local variations in the incidence of HCC within Inhambane Province (Harington *et al.*, 1975) and the preliminary survey was therefore expanded into a programme lasting 7 years in order to accumulate sufficient data for assessment of regional variation of HCC in relation to aflatoxin contamination within the Province. The detailed regional information is given in the present paper. Studies of aflatoxin contamination were also made in Manhica-Magude District, an area just to the north of Maputo, where the mineworkers' survey indicated a low incidence of HCC, and in Transkei, situated some 500 km south of Mozambique, where a survey of cancer incidence was being undertaken (Rose & Fellingham, 1981).

*Present address: I.C.I., Central Toxicology Laboratory, Alderley Park, Macclesfield, SK10 4TJ, Cheshire, UK.
Correspondence: P. Cook-Mozaffari

Received 13 October 1984; and in revised form 21 January 1985.

© The Macmillan Press Ltd., 1985

### Materials and methods

### *Incidence of HCC*

*Mozambique – The Inhambane Survey* The region investigated encompassed most of Inhambane Province, which is a coastal Province approximately midway between the towns of Maputo and Beira (Figure 1). It is subdivided into 10 districts of which two in the remote north (Govuro and Vilanculos) were excluded from the study since there were no hospitals within a reasonable distance which could provide an adequate standard of diagnosis for HCC. The remaining eight districts occupied an area of 38,656 square kilometres.

Histological confirmation of hepatocellular carcinoma was the primary requirement before registration was considered. Needle biopsies were performed throughout the period of the survey giving a relatively easy method of accurate diagnosis. For each patient basic personal information was recorded, viz: name, sex, age, home location and the date when the biopsy was taken.

More than 90% of all cases recorded were registered at the Chicuque Hospital which is situated in the Maxixe district. This hospital, under the direction of Dr R.L. Simpson, was well-known in the area for its standard of care. Several other small hospital/clinics existed throughout the study area, but none had resident doctors, with the exception at times of the hospital in the town of Inhambane a few kilometres across the bay from Chicuque, where a few patients qualified for registration.

Registration commenced in February, 1968 and continued for 7 years to the end of January, 1975. In May 1970, in the third year of registration, a national census was conducted which gave an age, sex breakdown of the population for individual districts of Inhambane Province (Instituto Nacional De Estatistica, 1974).

Numerous young men from Mozambique work

**Figure 1** Map of southern Africa, indicating places mentioned in the text. Blackened areas indicate locations of dietary aflatoxin studies which were performed in Kenya (Peers & Linsell, 1973), Swaziland (Peers *et al.*, 1976), Transkei and within Mozambique the Inhambane Province and the Manhica-Magude region (*this report*).

at the gold mines in Johannesburg where they are under constant medical surveillance. Incidence rates for HCC among gold mineworkers from different regions of southern Africa, including different districts within Mozambique, have been published for the years 1964 to 1979 (Harington *et al.*, 1975; Bradshaw *et al.*, 1982). The census statistics for Mozambique are said to include the men who are temporarily absent. However, the hospital staff who provided the cancer data for the present survey were relatively confident that biopsies, which would qualify these workers for registration, were not taken from mineworkers sent home after diagnosis. In calculating incidence rates, therefore, the average number of men absent at the mines (Bradshaw *et al.*, 1982) has been subtracted from the district populations. The majority of absent workers from Inhambane Province were employed in the South African gold mines so that serious bias will not have been introduced by failure to take account of people who were employed in other industries. Miners are recruited between the ages of 18 and 40 but men are re-engaged after the age of 40 provided that they are fit for work (Harington *et al.*, 1975). For the present survey, it has been assumed that the miners were divided between the age groups 20–29; 30–39 and 40–49 in the proportions 40%, 40% and 20%.

Age-specific and age-standardised rates of HCC have been calculated for Inhambane and its districts using the "African Standard Population". In order to obtain data for comparison with aflatoxin exposure studies performed elsewhere in Africa and Asia crude rates have also been calculated.

A fall in HCC rates among workers from Mozambique has been observed in successive stages of the mineworkers survey (Bradshaw & Harington, 1976; Bradshaw *et al.*, 1982). In view of this, crude incidence rates for the pooled data from the eight Inhambane districts have been calculated for individual years using population estimates that were derived by linear interpolation and extrapolation from the 1960 and 1970 census reports.

*Mozambique – Manhica-Magude district* Manhica-Magude district, lying somewhat to the south of Inhambane Province, was included in the food sampling programme for the assessment of aflatoxin levels because of the very low rates for HCC indicated in the mineworkers survey (Harington *et al.*, 1975). It was not covered by the local cancer registration schemes. Estimates of age-standardised incidence have therefore been derived by comparing the results of the mineworkers survey for Manhica-Magude with the pooled results for the Inhambane districts. A crude annual rate of 38.4 was observed for the mineworkers from Manhica-Magude and a crude rate of 81.1 for the mineworkers from Inhambane. The pooled age-standardised rates and crude total rates for Inhambane Province have been multiplied by 38.4/81.1 to give estimates of rates for Manhica-Magude district.

*South Africa – Transkei* Incidence rates for HCC in Transkei are available for the period 1965 to 1969 (Rose & Fellingham, 1981). Rates were calculated using the African standard population, as in Mozambique. Absent mineworkers were not included in the census returns for the Transkei and it is thought (Rose & Fellingham, 1981) that HCC patients diagnosed at the mining hospitals were not included in the cancer returns from Transkei. The problem of adjusting for the absent mineworkers does not, therefore, arise in the Transkei survey.

The incidence rates for the Transkei are for an earlier period than that during which food samples were collected for aflatoxin analysis, whereas in Mozambique the incidence and food analysis surveys took place contemporaneously. In order, therefore, to examine the stability of rates over time in Transkei, information was again sought from the mineworkers survey. For the period 1964–71, 71 patients diagnosed amongst mineworkers from Transkei yielded a rate of 12.9 per 100,000 man years (Harington *et al.*, 1975). The period 1972–79 saw 65 cases with a rate of 12.8 per 100,000 man years (Bradshaw *et al.*, 1982). It would seem, therefore, that HCC rates in Transkei, unlike those in Mozambique, have remained fairly stable over the years.

*Assessment of aflatoxin contamination of food*

*Regions investigated* Food samples were collected between 1969 and 1974 from seven of the eight districts included in the cancer registration programme within Inhambane Province. The district of Panda was not covered by the sampling programme and, for estimates of contamination levels, the small district of Maxixe was combined with Homoine District. Samples were also obtained from Manhica-Magude District. For convenience, sampling in Transkei took place in four districts which had previously been selected for studies on the occurrence of cancer of the oesophagus (Bizanna, Lusikisiki, Kentani and Butterworth) (Rose, 1973). The combined incidence of HCC for these four districts was very similar to the rates for the whole Transkei and it was, therefore, assumed that the study regions were representative of the whole territory. Samples were collected during 1976 and 1977. The number of patients with HCC and the number of food samples taken in Transkei were not sufficient for analysis by individual districts and the four areas were taken as a single study region.

*Sampling procedures* The field procedure in Mozambique was to approach all those headmen throughout the study area whose villages were reasonably accessible and to obtain their co-operation in collecting several 100 g samples of the main meal of the day from surrounding home-steads. Samples were weighed as soon as they were removed from the cooking pot. The varied dietary habits of the region and the ingredients most liable to contamination have been described in a previous paper (Van Rensburg *et al.*, 1975). In general, meals consist of three basic ingredients (a carbo-hydrate staple, a protein-rich food and a green vegetable).

In Transkei an individual who was unfamiliar with the region marked collection points on a map along the well developed network of roads to give an approximate grid coverage of each district. The field team arrived unannounced and obtained a 100 g cooked-food sample from the nearest house-hold.

The diet of Transkei is more monotonous than that of Inhambane. The staple of the region is maize which is the basic ingredient of 95% of meals, about half of which are supplemented by means or vegetables such as pumpkin or "wild spinach" (Groenewald *et al.*, 1981). In view of the very low intake of animal protein. no attempt was made to sample the rare meat dishes encountered.

*Aflatoxin analysis* In the field, samples were mixed with 20 ml chloroform immediately after they had been weighed and then were stored at 5°C until assayed.

In the laboratory, the whole of each sample was mixed with 250 ml chloroform and 50 ml water, and homogenised in a Sorvall blender at maximum speed for 2 min. After centrifugation at 2,500 r/min for 15 min, the extract was filtered and the chloro-form phase collected quantitatively. Further clean-up procedures, silica gel 60 column chromato-graphy and thin layer chromatography were performed as described by Peers & Linsell (1973). Confirmatory derivative formation (Przybylski, 1975) was always performed when adequate aflatoxin $B_1$ was present ($>5\,\mu g\,kg^{-1}$).

### Results (i)

*Incidence of HCC*

In the Inhambane survey a total of 493 HCC patients were registered amongst 581,667 people who lived in the study area. Census records showed that 98.8% of all the people in the Inhambane Province were blacks and all HCC patients registered belonged to this ethnic group.

Male incidence rates ranged from 9.3 to 60.7 per 100,000 (Table I) and female incidence rates varied from 3.7 to 13.0 per 100,000. Rates are high for both sexes in the inland district of Panda and low for both sexes in the northern region of Massinga (Figure 2). They are high for men (47.9 per 100,000) and moderately high for women (10.3 per 100,000) in the combined districts of Homoine-Maxixe.

The rates recorded among mineworkers from Inhambane Province (Bradshaw *et al.*, 1982) are

**Table I** Age-standardised incidence of HCC per 100,000 in eight districts of Inhambane Province, Mozambique (1968–74) and in Transkei (1965–69) (rates adjusted to the African standard), and estimated rates for Manhica-Magude District, Mozambique

| | *Population* | | *HCC cases* | | *Incidence rates* | | |
|---|---|---|---|---|---|---|---|
| | *Total* | *Density* ($km^{-2}$) | *Males* | *Females* | *Males* | *Females* | *Mine-workers*[a] |
| Massinga | 148403 | 7.3 | 35 | 15 | 9.3 | 3.7 | 56.2 |
| Morrumbene | 75579 | 27.0 | 48 | 31 | 29.1 | 12.1 | 65.3 |
| Homoine-Maxixe | 109887 | 49.4 | 92 | 38 | 47.9 | 10.3 | 58.4 |
| Panda | 35097 | 4.9 | 38 | 15 | 60.7 | 13.0 | 123.4 |
| Inhambane | 83980 | 56.7 | 46 | 23 | 21.8 | 9.2 | 110.1 |
| Inharrime | 57278 | 22.3 | 23 | 12 | 17.8 | 6.7 | 113.2 |
| Zavala | 81443 | 41.4 | 48 | 29 | 28.8 | 13.0 | 111.8 |
| Total Inhambane Districts | 591667 | | 330 | 163 | 25.5 | 8.9 | 81.1 |
| Transkei | — | — | 262 | 116 | 9.1 | 2.2 | 11.4 |
| Manhica-Magude | — | — | — | — | 12.1 | 4.2 | 38.4 |

[a]Rates for 1964–79 (Bradshaw *et al.*, 1982), crude rates for men mostly aged between 20 and 50.

**Figure 2** Districts of the Inhambane Province of Mozambique showing age-standardized HCC rates for each sex (African standard).

shown in Table I alongside the local rates. These also indicate a high rate in Panda and a low rate in Massinga but the ranking of the other districts differs. In particular Homoine-Maxixe District had one of the lowest incidence rates within Inhambane Province in the mineworkers survey.

Age-specific rates for the combined eight districts of Inhambane Province are presented in Table II. Similar age-specific rates for other centres in Africa have been calculated from data given in the first two volumes of "Cancer Incidence in five Continents" (Doll *et al.*, 1966, 1970) to give values for the same age groups as have been used in the Inhambane survey and these are plotted in Figure 3 in comparison with the Inhambane figures. The places for which rates are given are shown in Figure 1. Age-specific rates for Taiwan (Beasley *et al.*, 1981), England and the USA (Waterhouse *et al.*, 1976, 1982) are also presented in Figure 3. In using the data from Africa the categories for "primary liver cancer" and "liver, secondary and unspecified" (WHO International Classification of Disease, 7th edition, 155.0 and 156) have been combined. The only two centres where an appreciable number of cases is added by inclusion of category 156 are Kyadondo, Uganda and Ibadan, Nigeria. In both centres, cancers at the sites which normally metastasise to the liver are rare and so the "unspecified" cases are most likely to be primary tumours. In the West where the incidence of HCC is low and where sites that metastasise to the liver are overwhelmingly more common it has in the past been difficult to establish the true incidence of HCC at older ages (D. Zaridze and R. Doll, personal communication). The rates for the USA shown in Figure 3 are based on recent cancer registry data in which there was a high level

**Table II** Age specific rates by district and sex, Inhambane Province, Mozambique

| | *Massinga* | *Morrumbene* | *Homoine and Maxixe* | *Panda* | *Inhambane* | *Inharrime* | *Zavala* | *Combined districts rate* | *no.* |
|---|---|---|---|---|---|---|---|---|---|
| Males | | | | | | | | | |
| 0– | 0.0 | 2.7 | 1.9 | 3.0 | 0.0 | 0.0 | 0.0 | 0.8 | 5 |
| 10– | 0.9 | 9.5 | 14.4 | 22.0 | 6.3 | 5.7 | 6.9 | 7.5 | 31 |
| 20– | 9.5 | 42.0 | 91.5 | 123.2 | 30.0 | 40.6 | 68.5 | 43.5 | 91 |
| 30– | 14.0 | 58.7 | 94.2 | 95.7 | 48.1 | 25.2 | 40.4 | 45.4 | 83 |
| 40– | 21.0 | 38.1 | 53.4 | 78.6 | 28.5 | 8.5 | 22.6 | 33.4 | 53 |
| 50– | 31.5 | 34.7 | 16.6 | 72.0 | 31.5 | 25.1 | 47.8 | 33.2 | 45 |
| 60– | 23.1 | 34.2 | 25.5 | 16.1 | 16.7 | 7.4 | 16.0 | 20.0 | 19 |
| 70+ | 0.0 | 0.0 | 27.6 | 0.0 | 0.0 | 60.8 | 24.9 | 20.8 | 3 |
| Females | | | | | | | | | |
| 0– | 0.0 | 0.0 | 2.8 | 0.0 | 0.0 | 0.0 | 0.0 | 0.5 | 3 |
| 10– | 2.7 | 2.0 | 7.1 | 9.4 | 10.8 | 3.4 | 2.1 | 5.2 | 18 |
| 20– | 6.2 | 9.1 | 18.8 | 27.5 | 13.7 | 0.0 | 30.5 | 13.6 | 44 |
| 30– | 6.3 | 28.7 | 15.5 | 17.6 | 14.2 | 20.1 | 21.2 | 15.9 | 49 |
| 40– | 4.8 | 25.6 | 10.2 | 17.2 | 7.1 | 10.1 | 14.0 | 12.1 | 27 |
| 50– | 0.0 | 14.7 | 5.6 | 8.6 | 9.4 | 18.8 | 8.9 | 8.7 | 15 |
| 60– | 5.3 | 28.4 | 4.0 | 0.0 | 10.0 | 0.0 | 0.0 | 5.2 | 6 |
| 70+ | 0.0 | 0.0 | 0.0 | 0.0 | 0.0 | 0.0 | 17.9 | 4.5 | 1 |

**Figure 3** Age-specific incidence rates of HCC for Inhambane Province and for other centres in Africa compared with the incidence in the UK (Birmingham 1968–72, S. Metropolitan Region 1967–71, Liverpool 1968–72) and the USA (White populations of Alameda County, California, San Francisco Bay Area, Los Angeles, Atlanta, New Orleans, Connecticut and Detroit for the period 1973–77).

of histological confirmation of diagnosis even at older ages.

Remarkably high rates in young persons (aged 10–19 and 20–29) are an outstanding feature of the Inhambane Province rates. Moreover the incidence in the age groups from 20–29 to 50–59 is at a similar level without the gradient of increasing incidence with age that is commonly found elsewhere in the world. The age curves for Maputo have a similar shape but those for other areas of the world all show a more or less linear increase when the logarithm of incidence is plotted against the logarithm of age (Figure 3).

The ratio of male to female incidence in Inhambane Province varies from around 1.5 to one in the age groups 0–9 and 10–19, to three to one in the age groups 20–29, 30–39 and 40–49 and increases to four or more to one in the three oldest age groups.

Age-specific rates for individual districts in Inhambane Province are also shown in Table II. In most districts, the age-specific rates for men between the ages of 20 and 49 are lower than the rates indicated for this age group in the mine-workers survey (shown here in Table I).

The calculations for crude incidence rates for each year of the Inhambane survey indicate a relatively smooth fall in incidence from year to year and a decrease of 43% between 1968 and 1973, from a rate of 17.4 to 9.9 per 100,000. The regression of incidence against year indicated a significant decline during this period ($P<0.05$). The rate dropped further to 6.4 during 1974 but during this time there were periods when the principal hospital of the survey was understaffed.

### Discussion (i)

### *HCC incidence*

*Variation between districts of Inhambane Province*
Results have been presented that indicate marked regional variation in frequency within Inhambane Province. However, comparison of the rates calculated from the local survey with those of the mineworkers survey (Bradshaw *et al.*, 1982) suggest that the rates recorded locally are lower than might be expected and indicate discrepancies in the ranking of individual districts. It is probable that the difference between the two surveys arise largely, although not entirely, from a decreasing attendance at hospital with increasing distance from the hospital where the majority of patients were seen in the local survey. It has long been established that in Africa attendance at hospital falls rapidly with distance from the patient's home (Davies *et al.*, 1965; Jolly & King, 1966). The availability of an independent set of rates from the mineworkers survey, that would not be subject to this bias, permits an estimation of the degree of under-reporting that has occurred within Inhambane Province.

The indications for underreporting in the local Inhambane Province data are as follows. The greatest differences between the rates recorded locally for men aged 20–49 (Table II) and the rates observed in the mineworkers survey (shown here in Table I) are three to four-fold in extent and occur in the districts that are furthest from Chiquque hospital, – that is in the districts of Massinga, Inharrime and Zavala, – while the smallest differences, near to unity, are in the districts adjacent to the hospital, – in Homoine-Maxixe and in Morrumbene. Furthermore the rates estimated for men and women in Manhica-Magude District (Table I) (estimates based on the combined 8-district rate for Inhambane Province that was observed locally) are only slightly above the rates recorded in the Transkei (Rose & Fellingham, 1981) whereas the mineworkers survey indicated a three-fold difference in rate between Manhica-Magude and Transkei. Both points suggest local underreporting in the Inhambane survey. Conversely, the fact that the rates observed locally in Homoine-Maxixe for men aged 20–29 and 30–39 are actually slightly above the rate recorded in the mineworkers survey may mean that some patients are giving the address of relatives who live near the hospital and that the rate for this district is to some extent overreported in the local survey.

In part, the discrepancy between the two surveys may reflect problems in allocating addresses correctly in the mineworkers survey (conducted over 500 miles away in a different country). In particular the high rate recorded for Inhambane District in contrast to the low rate in the local survey may represent confusion between Inhambane District and Inhambane Province.

The highest and lowest rates occur in the same districts in the two surveys but doubt remains as to the precise ranking of the districts and, in subsequent comparisons with levels of aflatoxin contamination, use has, therefore, been made both of the local rates and of the rates from the mineworkers survey.

When considering the age-specific rates for Inhambane Province as a whole, the information from the mineworkers survey permits an adjustment for underreporting that is less uncertain than those for individual districts. A rate of 81.1 per 100,000 was recorded for the mineworkers in the combined eight districts of Inhambane (rate adjusted to allow for the differing proportion (6 to 13%) of the male population who were absent from the mines from each district). This compares with rates of just over 40 per 100,000 in the local survey for men of comparable age (Table II). Assuming that the miners are divided in the proportion 40%, 40% and 20% between the age groups 20–29, 30–39 and 40–49 (i.e. the assumption made above in subtracting the mining populations from the local populations' and assuming that the HCC cases among the miners are divided so as to give age-specific rates proportional to those observed locally in the combined Inhambane Districts (Table II), then the estimated rates among the miners for these age groups would be 82.2, 85.8 and 63.1, – giving the crude rate of 81.1 noted above and indicating a 1.9-fold increase over the local rates.

Given that underreporting of this extent has almost certainly occurred in the Inhambane survey it could be that the falling away from the expected shape of age curve for HCC patients noted in the local rates (Figure 3) simply represents progressively greater under-attendance at hospital with increasing age. There are several pieces of evidence, however, which suggest that this is not the principal explanation. In the first place, the flattening of the curves for both men and women starts at a relatively young age (i.e. in the age group 30–39). Secondly the most striking feature of Figure 3 is the similarity between the shape of age curves for Inhambane and those for Maputo. For Maputo, the pooled age-specific incidence has been calculated also for 13 sites of cancer* which would be expected, on a double logarithmic scale, to show an approximately linear relationship between incidence and age (Cook *et al.*, 1969). There is no indication in these figures of the gross departure from linearity that is apparent for the HCC curves and that would occur if the latter were due merely to under-attendance at hospital (Figure 4). Unfortunately no figures for other types of cancer exist for Inhambane but it can be assumed that the pattern of attendance is not very dissimilar from that in Maputo. In part, of course, the shape of the age curve for men in Inhambane is determined by the assumptions concerning the age of the absent mineworkers but only to a limited extent. Little difference is made if the assumptions are changed so as to postulate alternative age structures at the limit of likelihood in either direction. A final piece of evidence suggesting that the shape of age curve observed at the two centres in Mozambique is probably genuine comes from the mineworkers survey (Harington *et al.*, 1975). The patients with liver cancer had a younger age structure than the patients with bladder cancer. The majority of patients in both groups (487/710 and 43/65 came from Mozambique.

The ratio of male to female incidence at all ages in Inhambane Province (Table II) is higher than that in the UK or the USA. The increase in the ratio for the age groups over the age of 50 in Inhambane probably does represent a greater tendency for older women not to attend hospital.

*Cancers of the mouth, nasopharynx, other pharynx, oesophagus, stomach, colon, rectum, pancreas, larynx, bronchus and trachea, penis, bladder and skin (other than melanoma).

**Figure 4** Age-specific incidence rates for epithelial carcinomas other than HCC for Maputo (....), Natal (----) and Birmingham, UK (——). The rates include carcinomas located in the mouth, nasopharynx, other pharynx, oesophagus, stomach, colon, rectum, pancreas, larynx, bronchus-trachea, penis, bladder and skin (other than melanomas).

*Decline in incidence with time* The decline in incidence of HCC of 43% between 1968 and 1973 in the Inhambane survey is consistent with the results of the mineworkers survey. Between the two periods for which the mineworkers data are available (1964–71 and 1972–79) there was a decline of 37% among mineworkers from the combined 8 Inhambane districts included in the local survey. Among mineworkers from elsewhere in Mozambique there was a decline of 44% (calculations from data presented in Harington *et al.,* 1975 and in Bradshaw *et al.,* 1982). The declining incidence in Mozambique is slightly more than twice the decrease (18%) that has occurred among mineworkers from other parts of southern Africa where the rate has gone down from 10.1 to 8.3 per 100,000.

### Results (ii)

*Contamination of food by aflatoxin in relation to HCC incidence*

Some 8% of the 2183 prepared meal samples in Mozambique contained measurable amounts of aflatoxin (Table III). Aflatoxin $B_1$ constituted 89.1% of the total aflatoxin measured and aflatoxin $B_2$ 6.4% with lesser amounts of $G_1$ and $G_2$. The average contamination level for $B_1$ of positive samples was 38.1 $\mu g\,kg^{-1}$ wet food and the highest contamination level of a single sample was 1517 $\mu g\,kg^{-1}$ in a meal consisting mainly of groundnuts. The average consumption level for aflatoxin $B_1$ for all samples was 2.9 $\mu g\,kg^{-1}$ and within Mozambique there was 9-fold variation in the mean district contamination values for all samples (Table IV).

The percentage of samples found to contain aflatoxin was much higher in Transkei than in Mozambique (Table IV) but the concentrations were exceedingly low and only $B_1$ was detected. The highest value for a maize-based meal was 10.1 $\mu g\,kg^{-1}$ and this was exceeded only by a sample of soured milk that contained 27.3 $\mu g\,l^{-1}$. Food samples taken over two seasons in Transkei yielded similar results in both seasons; an overall $B_1$ contamination rate of 0.65 $\mu g\,kg^{-1}$ wet food in 1976 and 0.66 $\mu g\,kg^{-1}$ in 1977.

**Table III** Occurrence of individual aflatoxins in prepared food samples from Mozambique (2183 meals)

| | *Aflatoxin* | | | |
|---|---|---|---|---|
| | $B_1$ | $B_2$ | $G_1$ | $G_2$ |
| Percentage of samples positive | 8.0 | 2.8 | 0.8 | 0.7 |
| Percentage of total aflatoxin | 89.1 | 6.4 | 3.5 | 1.0 |
| Mean contamination of positive samples ($\mu g\,kg^{-1}$) | 38.1 | 7.4 | 13.7 | 4.4 |
| Mean value for all samples[a] ($\mu g\,kg^{-1}$) | 2.90 | 0.21 | 0.11 | 0.03 |

[a]This overall value includes all samples where no aflatoxins were detected as 0.

**Table IV** Aflatoxin contamination of food in Transkei and in seven districts of Mozambique

| District | Total samples | % positive | Aflatoxin conc. ($\mu g\,kg^{-1}$ wet food) $B_1$ | Total |
|---|---|---|---|---|
| Transkei | 623 | 24.7 | 0.66 | 0.66 |
| Manhica-Magude | 504 | 4.3 | 0.71 | 0.82 |
| Massinga | 247 | 2.4 | 1.35 | 1.35 |
| Morrumbene | 304 | 7.2 | 3.07 | 3.13 |
| Inharrime | 261 | 5.2 | 3.04 | 3.50 |
| Inhambane | 307 | 11.7 | 2.72 | 3.97 |
| Homoine-Maxixe | 291 | 13.4 | 4.60 | 5.12 |
| Zavala | 269 | 11.9 | 6.43 | 6.62 |

Only a few samples of home brewed maize beer were taken since representative sampling proved difficult. However only 6 of the 23 samples that were tested were positive and these had uniformly low concentrations of aflatoxin resulting in an overall average $B_1$ contamination of $0.69\,\mu g\,l^{-1}$. There is no evidence, therefore, that the omission of alcoholic drinks from the estimations of average contamination levels will have biased results.

Rank correlation co-efficients (Kendal's T) have been calculated for the association between liver cancer incidence and aflatoxin contamination for the six districts of Inhambane for which contamination data are available plus Manhica–Magude district and Transkei (Table V). For men both the local rates and the mineworkers rates have been used. The association is significant for both men and women and for both aflatoxin $B_1$ and total aflatoxin. For both measures of contamination, the association for women is stronger than either of the associations for men.

In Table VI the Mozambique and Transkei results are added to those from other parts of Africa and Asia. The incidence rate that has been derived for all the areas for which aflatoxin data are available is a crude rate using pooled male and female figures (Van Rensburg, 1977; Linsell & Peers, 1977). The crude rates for Inhambane districts shown in Table VI are based on the cancers diagnosed locally and have been calculated in the same way as were the age-standardised rates shown in Table I (i.e. with the mining population subtracted from the total male population). Calculation of correlation-co-coefficients shows a highly significant association between aflatoxin $B_1$ intake and the level of HCC incidence; between the logarithm of aflatoxin $B_1$ intake and HCC incidence and between the logarithm of aflatoxin $B_1$ intake and the logarithm of HCC incidence. The value of "r" is lowest for the simple linear association and highest for the double logarithmic association. Figure 5 shows the plot of incidence against intake on a double logarithmic scale.

**Table V** Rank correlation co-efficients between HCC incidence and aflatoxin intake in seven districts of Mozambique and in Transkei

| | Aflatoxin $B_1$ | Total aflatoxin |
|---|---|---|
| Males – age-standardised rates | 0.71[b] | 0.64[a] |
| – mine-workers' rates (Bradshaw *et al.*, 1982) | 0.57[a] | 0.64[a] |
| Females – age-standardised rates | 0.79[b] | 0.71[b] |

[a]$P<0.05-0.01$.
[b]$P<0.01-0.001$.

**Figure 5** Available combined data demonstrating the relationship between dietary aflatoxin $B_1$ exposure and the HCC rate on a double logarithmic scale ($r=0.95$; $P=<0.001$). (○) Kenya; (□) Thailand; (X) Mozambique; (●) Swaziland; (■) Transkei.

### General discussion

The occurrence of HCC in Mozambique is characterized by six features which require explanation:

1. An exceptionally high incidence by world standards.
2. Geographic variation between neighbouring regions.
3. A very high incidence in adolescents and young adults which is not sustained at older ages.
4. A decline in incidence with time during the period for which figures are available in the last three decades.
5. A higher incidence in men than women.
6. A changing sex ratio with age.

The last two features of the occurrence of HCC in Mozambique are common also in other parts of the world (Doll *et al.*, 1970; Waterhouse *et al.*, 1976) and will not be considered further in the present paper.

**Table VI** Summarised results of studies measuring crude HCC rate (males and females) and aflatoxin intake ($ng\,kg^{-1}$ body wt $day^{-1}$) with cooked food

| | | Locale | HCC rate ($10^5$/year) | Aflatoxin $B_1$ intake |
|---|---|---|---|---|
| Kenya | : | High altitude | 1.2 | 3.5 |
| Thailand | : | Songkhla | 2.0 | 5.0 |
| Swaziland | : | Highveld | 2.2 | 5.1 |
| Kenya | : | Middle altitude | 2.5 | 5.9 |
| Swaziland | : | Middleveld | 3.8 | 8.9 |
| Kenya | : | Low altitude | 4.0 | 10.0 |
| Swaziland | : | Lebombo | 4.3 | 15.4 |
| Thailand | : | Ratburi | 6.0 | 45.0 |
| Transkei | : | Four districts | 6.9 | 16.5 |
| Mozambique | : | Manhica-Magude | 5.9 | 20.3 |
| Swaziland | : | Lowveld | 9.2 | 43.1 |
| Mozambique | : | Massinga | 5.0 | 38.6 |
| Mozambique | : | Inharrime | 9.0 | 86.9 |
| Mozambique | : | Inhambane | 12.1 | 77.7 |
| Mozambique | : | Morrumbene | 15.5 | 87.7 |
| Mozambique | : | Homoine-Maxixe | 17.7 | 131.4 |
| Mozambique | : | Zavala | 14.0 | 183.7 |

HCC incidence *vs* aflatoxin intake: $r=0.8792$, $P<0.001$.
HCC incidence *vs* the log of aflatoxin intake: $r=0.9051$, $P<0.001$.
Log HCC incidence *vs* log aflatoxin intake: $r=0.9480$, $P<0.001$.

Three major factors have been identified in different parts of the world as apparently having a causal role in the development of HCC: the ingestion of foodstuffs contaminated by aflatoxin; chronic active infection with HBV, and an excessive intake of alcohol (recent reviews of all factors are given by Falk, 1982 and by Munos & Linsell, 1982). Various industrial chemicals (Hoover & Fraumeni, 1975) and the contraceptive pill (Henderson *et al.*, 1983; Forman & Doll, 1983) may be responsible for a few cases in countries where these are used extensively and the smoking of cigarettes may give some increase in risk (Trichopoulos *et al.*, 1980). There is very little evidence to indicate that alcohol has played a role in the high incidence areas of HCC in Africa. A case-control study in South Africa gave no indication that it was implicated as a risk factor (Higginson & Oettlé, 1960) and until recently cirrhosis associated with an excessive intake of alcohol has rarely been seen in HCC patients from Africa (Higginson, 1956; Maynard *et al.*, 1970; Van den Heever *et al.*, 1978). The smoking of cigarettes has only recently started to spread in rural Africa and its contribution, if any, to be the very high rates of HCC observed in Mozambique can only be negligible. The same is true for industrial chemicals and oral contraceptives, so that the major factors which remain for consideration are aflatoxin and chronic HBV infection.

Data have been given in the present paper which indicate that variations in the level of ingestion of aflatoxin could be the principal cause of both the exceptionally high incidence of HCC in Mozambique relative to the rest of the world and of the differences that have been observed between regions within Mozambique. The aflatoxin levels observed in food samples are the highest ever recorded in any part of the world and various methods of looking at the data all suggest a geographical association with HCC incidence both locally and for all the areas within Africa and Asia for which reliable estimates of aflatoxin intake are available. A similar correlation between aflatoxin intake and age-standardised HCC mortality rates has recently been reported from China where there is considerable geographical variation in the occurrence of HCC with loci of very high incidence (Yeh *et al.*, 1982). Further confirmation of an aetiological role for aflatoxin has also come from a case-control study in the Philippines where the mean contamination level of different dietary items was established and individual levels of consumption determined retrospectively (Bulatayo–Jayme *et al.*, 1982) although no such association is apparent in a case-control study conducted in Hong Kong (Lam *et al.*, 1982).

The geographical pattern of incidence observed in Inhambane, where a higher incidence occurs in the drier, inland transitional savanna area of Panda than in the wetter forested area along the coast, is more like that reported from the Ivory Coast of West Africa (Tuyns *et al.*, 1971) than the situation observed in Kenya and Swaziland where incidence was high in the hot humid areas of lower altitude and less high in the colder though wetter areas of higher altitude. The Inhambane findings may reflect the fact that aflatoxin contamination of peanuts grown in southern Africa is invariably higher in lower rainfall regions, due to factors such as premature death of nuts and husk damage by moisture-seeking termites.

The numerous case-control studies that indicated a causative role for HBV infection in the development of HCC (Tabor *et al.*, 1977; Maupas *et al.*, 1977; Kubo *et al.*, 1977; Johnson *et al.*, 1978; Trichopoulos *et al.*, 1978; Kew *et al.*, 1979; Omata *et al.*, 1979) have been followed by evidence that suggests that chronic active infection with HBV may be a necessary initial factor in the development of all cases of HCC. A prospective study in Taiwan has indicated a relative risk of over 200 with all but one case occurring among persons who were carriers prior to development of HCC (Beasley *et al.*, 1981) while viral DNA has been found incorporated into the host genome of HCC patients

(Shafritz *et al.*, 1981) including those who have no viral markers of HBV infection (Brechot *et al.*, 1982).

It has also been suggested that chronic HBV infection acting alone could be responsible for the large range of incidence for HCC that exists between different countries and that aflatoxin has little carcinogenic effect on humans (Prince, 1978; Lutwick, 1979). This hypothesis was based on broad geographical comparisons of the two diseases and on estimation of a similar risk for HCC among carriers of HBV in Mozambique and in the USA.

If chronic HBV infection were indeed a sufficient cause for the development of HCC, a close geographical association between the two diseases would be expected, whereas several regions of anomalously high or low incidence for HCC relative to the occurrence of HBV have been reported. Bagshawe *et al.* (1975) found no difference in the prevalence of HBsAg in areas where an association had been demonstrated between HCC incidence and aflatoxin contamination (Peers & Linsell, 1973). Ziegler *et al.* (1977) drew attention to an apparent low incidence of HCC in Egypt despite a relatively high prevalence of HBsAg and a similar lack of association has been reported from North Africa, the Middle East and the Asian Republics of the Soviet Union (Szmuness, 1978). Prevalences of HbsAg as high as anywhere in Africa or Asia have been reported from Greenland (Skinhøj, 1977) and yet there is no evidence for an elevated risk of HCC above the low level that is found in neighbouring European or North American territories (Skinhøj *et al.*, 1978). Furthermore, higher prevalence of HbsAg have been reported from Taiwan both in control populations and among HCC patients (Tong *et al.*, 1971; Sung *et al.*, 1980) than have been found in Mozambique (Bersohn *et al.*, 1974; Kew *et al.*, 1979) and yet the age-standardised incidence for HCC is lower in Taiwan (calculations from data given in Beasley *et al.*, 1981) than in Mozambique. The one clear indication of a geographical association comes from Greece, an area of moderate incidence for HCC, where mortality rates from HCC in different regions showed a correlation with the proportion of army recruits who were positive for HBsAg in both rural and urban areas (Trichopoulos *et al.*, 1976).

No measurements of the prevalence of carrier status for HBV have been made for individual districts of Inhambane Province. However, data are available for blood donors from among the mine-workers from different countries and territories of southern Africa, including Mozambique (Bersohn *et al.*, 1974; Reys *et al.*, 1977) and these can be compared with the HCC incidence rates that have been observed for the same populations (Harington *et al.*, 1975). Estimations of the incidence of HCC among carriers of HBV made from these data (Cook–Mozaffari & Van Rensburg, in preparation) show a wide range of variation between different areas of southern Africa. The lack of a geographical association between the incidence of HCC and the prevalence of HBV carrier status is in striking contrast to the association which has been demonstrated above between HCC incidence and aflatoxin contamination within Asia and Africa.

The precise roles of HBV infection and aflatoxin intake in the development of HCC remain to be elucidated. The existence of regions of low incidence for HCC despite an elevated prevalence of HBV infection may indicate that HBV infection acting alone has little carcinogenic effect but that it needs to be potentiated by some other factor. The clear geographical association between aflatoxin contamination and HCC incidence in parts of Africa and Asia, despite the crude nature of the data, and the lack of an association with HBV prevalence at higher levels of incidence for HCC suggests that in these regions aflatoxin is a major determinant of risk. It is of interest that areas where a moderate to high prevalence of HBV is accompanied by a low incidence of HCC tend to be either too dry, as in Botswana, ot too cold, as in Greenland for the growth of aspergillus flavus. An aflatoxin survey in Egypt, which has a hot and dry climate, and a reportedly low incidence of HCC, revealed exceedingly low levels of contamination (Girgis *et al.*, 1977).

If, as the recent evidence cited above suggests, HBV infection is indeed a necessary initial factor in the development of HCC, it could be that, in Mozambique, where the intake of aflatoxin is exceptionally high but the prevalence of HBsAg is only moderate by African and Asian standards, the proportion of carriers in the population is actually a limiting factor in the occurrence of HCC and that this is the explanation of the abnormal shape of age curve for HCC that has been observed there in all districts except those of lowest incidence for HCC. Some susceptible individuals will of course be removed from the population by death from HCC. However, it is suggested that this is not the principal limiting factor, since only around one quarter of those who are positive for HBsAg will have died of HCC by the age of 40 (in the absence of other causes of death). Rather it is suggested that there are not enough susceptible individuals for the chance coming together of the two variables. Support is given to this hypothesis by the fact that aflatoxin contamination of foodstuffs within Mozambique would itself seem not be be uniform but to involve a relatively small proportion of the samples with some sporadically very high levels of contamination.

The existence of a pool of susceptible individuals would seem a more likely explanation of the unusual shape of age curve for HCG in Mozambique than would an especially high intake by young persons of contaminated dietary items. In regions where all food supplies are in relatively short supply it seems unlikely, for example, that children would have been permitted extra rations of a major dietary staple such as peanuts.

Just as it is postulated that a major determinant of variation in the incidence of HCC both within Mozambique and between Mozambique and other areas of the world is aflatoxin intake, so it would seem that a decline in intake levels may be responsible for the decrease in incidence that has occurred. Liver cancer is sufficiently common to be a familiar disease and there has been widespread publicity in Mozambique about the dangers of mouldy food which is likely to have resulted in increased selectivity during the preparation of foodstuffs. Also, as has happened with cashew nuts, economic pressures are probably reducing the consumption of peanuts, a crop that has been the most common dietary staple in Inhambane. High yielding and easily cultivated maize is, as in most of southern Africa, supplanting some traditional crops and, in Mozambique, maize does not seem as liable to serious contamination as peanuts or cassava (Van Rensburg *et al.*, 1975). If either hypothesis were correct it would suggest that aflatoxin was having a late stage effect in the development of HCC since both the dissemination of public health knowledge and the economic changes are recent phenomena. A further indication that aflatoxin has a late-stage effect comes from a survey among the South African gold miners (Purves, 1973) which indicated that after six months to a year at the mines the incidence of HCC declined quite sharply.* It can be assumed that the diet at the place of work contained very little aflatoxin since their commercial food supplies were under statutory control. If aflatoxin did indeed exert a late-stage effect, then intervention aimed at reducing consumption among HBV carriers would have good prospects of a reduction of HCC incidence within a few years.

In conclusion therefore, data have been presented from Mozambique and Transkei which indicate that the geographical association that has previously been noted between the contamination of foodstuffs by aflatoxin and the incidence of HCC in Africa and Asia continues at the highest levels of contamination and incidence. In Mozambique it appears that the level of incidence for HCC in middle and old age would be even higher were it not for a limitation probably imposed by an insufficient number of susceptible individuals in the general population. In view of the growing body of evidence that chronic active infection with HBV is a necessary first factor in the development of HCC, liver damage due to HBV is the presumed cause of susceptibility. The proportion of carriers that has been observed in Mozambique is only moderate by African and Asian standards and presumably is too low to allow the very high levels of aflatoxin that have been found to have their full carcinogenic potential. There are several indications from Mozambique that aflatoxin has a late-stage effect in the development of HCC, in which event the prospects for intervention would be good in persons who are already carriers for HBV.

*There is an employment turnover rate of almost 100% within 15 to 18 months (Purves, 1973) so that the incidence rates for the miners calculated over a period of years (Harington *et al.*, 1975; Bradshaw *et al.*, 1982) and used in the analysis above will not be greatly reduced by this effect.

### References

BAGSHAWE, A.F., GACENGI, D.M., CAMERON, C.H., DORMAN, J. & DANE, D.S. (1975). Hepatitis B antigen and liver cancer. *Br. J. Cancer*, **31**, 581.

BEASLEY, R.P., HWANG, L.Y., LIN, C.C. & CHIEN, C.S. (1981). Hepatocellular carcinoma and hepatitis B virus. *Lancet*, **ii**, 1129.

BERSOHN, L., MACNAB, G.M., PYZIKOWSKA, J. & KEW, M.C. (1974). The prevalence of hepatitis B (Australia) antigen in southern Africa. *S. Afr. Med. J.*, **48**, 941.

BRADSHAW, E., McGLASHAN, N.D., FITZGERALD, D. & HARINGTON, J.S. (1982). Analyses of cancer incidence in black gold miners from southern Africa (1964–79). *Br. J. Cancer*, **46**, 737.

BRADSHAW, E. & HARINGTON, J.S. (1976). Temporal changes in primary liver cancer in black goldminers from Mozambique. *S. Afr. Med. J.*, **50**, 2022.

BRECHOT, C., NALPAS, B. & COUROUCE, A. *et al.* (1982). Evidence that hepatitis B virus has a role in liver-cell carcinoma in alcoholic liver disease. *N. Engl. J. Med.*, **306**, 1384.

BULATAO-JAYME, J., ALMERO, E.M., CASTRO, Ma, C.A., JARDELEZA, Ma T.R. & SALAMAT, L.A. (1982). A case-control dietary study of primary liver cancer risk from aflatoxin exposure. *Int. J. Epidemiol.*, **11**, 112.

CARNAGHAN, R.B.A. (1967). Hepatitic tumours and other chronic liver changes in rats following a single oral administration of aflatoxin. *Br. J. Cancer*, **21**, 811.

COOK, P.J., DOLL, R. & FELLINGHAM, S.A. (1969). A mathematical model for the age distribution of cancer in man. *Int. J. Cancer*, **4**, 93.

DAVIES, J.N.P., KNOWELDEN, J. & WILSON, B.A. (1965). Incidence rates of cancer in Kyadondo County, Uganda, 1954–60. *J. Natl Cancer Inst.*, **35**, 789.

DOLL, R., PAYNE, P. & WATERHOUSE, J. (eds.) (1966). *Cancer Incidence in Five Continents.* Vol. 1, Berlin, Springer Verlag 219.

DOLL, R., MUIR, C., WATERHOUSE, J., (eds.). (1970). *Cancer Incidence in Five Continents.* Vol. 11 Berlin, Heidelburg, New York: Springer Verlag 388.

FALK, H. (1982). Liver. In: *Cancer Epidemiology and Prevention.* (Eds. Schottenfeld & Fraumeni) Philadelphia: W.B. Saunders p, 668.

FORMAN, D., DOLL, R. & PETO, R. (1983). Trends in mortality from carcinoma of the liver and the use of oral contraceptives. *Br. J. Cancer,* **48,** 349.

GIRGIS, A.N., EL-SHERIF, S., ROFAEL, N. & NESHEIM, S. (1977). Aflatoxins in Egyptian foodstuffs. *J. Ass. Off. Anal. Chem.,* **60,** 746.

GROENEWALD, G., LANGENHOVEN, M.L., BEYERS, M.J.C., DU PLESSIS, J.P., FERREIRA, J.J. & VAN RENSBURG, S.J. (1981). Nutrient intakes among rural Transkeians at risk for oesophageal cancer. *S. Afr. Med. J.,* **60,** 964.

HARINGTON, J.S., McGLASHAN, N.D., BRADSHAW, E., GEDDES, E.W. & PURVES, L.R. (1975). A spatial and temporal analysis of four cancers in African gold miners from Southern Africa. *Br. J. Cancer,* **31,** 665.

HENDERSON, S., PRESTON MARTIN, H.A., EDMONSON, R.L.P. & PIKE, M.C. (1983). Hepatocellular carcinoma and oral contraceptives. *Br. J. Cancer,* **48,** 437.

HIGGINSON, J. (1956). Primary carcinoma of the liver in Africa. *Br. J. Cancer,* **10,** 609.

HIGGINSON, J. & OETTLE, A.G. (1960). Cancer incidence in the Bantu and "Cape Coloured" races of South Africa. Report of a cancer survey in the Transvaal (1953–55). *J. Natl Cancer Inst.,* **24,** 589.

HOOVER, R. & FRAUMENI, J.F. (1975). Cancer mortality in US counties with chemical industries. *Environ. Res.,* **9,** 196.

INSTITUTO NACIONAL DE ESTATISTICA IV. (1974). Recenseamento geral da populacao – 1970. Direccao Provincial Dos Servicos De Estatistica, Lourenco Marques Vol. 7.

JOHNSON, P.J., KRASNER, N., PORTMANN, B., EDDLESTON, A.L.W.F. & WILLIAMS, R. (1978). Hepatocellular carcinoma in Great Britain: Influence of age, sex, HBsAg status, and aetiology of underlying cirrhosis. *Gut,* **19,** 1022.

JOLLY, R. & KING, M. (1966). The organisation of health services. In: *Medical Care in Developing Countries.* (Ed. King), O.U.P. London, 3: 1–2, 15.

KEW, M.C., DESMYTER, J., BRADBURNE, A.F. & McNAB, G.M. (1979). Hepatitis B virus infection in southern African blacks with hepatocellular cancer. *J. Natl Cancer Inst.,* **62,** 517.

KUBO, Y., KUNIO, O., MASAHARU, H. *et al.* (1977). Antibody to hepatitis B core antigen in patients with hepatocellular carcinoma. *Gastroenterology,* **72,** 1217.

LAM, K.C., YU, M.C., LEUNG, J.W.C. & HENDERSON, B.E. (1982). Hepatitis B virus and cigarette smoking: Risk factors for hepatocellular carcinoma in Hong Kong. *Cancer Res.,* **42,** 5246.

LINSELL, C.A. & PEERS, F.G. (1977). Aflatoxin and liver cell cancer. *Trans. Roy. Soc. Trop. Med. Nyg.,* **71,** 471.

LUTWICK, L.I. (1979). Relation between aflatoxin, hepatitis-B virus, and hepatocellular carcinoma. *Lancet,* **i,** 755.

MAPUS, Ph., COURSAGET, P., GOUDEAU, A., DRUCKER, J., *et al.* (1977). Hepatitis B virus and primary liver carcinoma: evidence for a filiation hepatitis B, cirrhosis and primary liver cancer. *Ann Microbiol.* (Inst. Pasteur), **128,** 245.

MAYNARD, E.P., SADIKALI, F., ANTHONY, P.P. & BARKER, L.F. (1970). Hepatitis associated antigen and cirrhosis in Uganda. *Lancet,* **ii,** 1326.

MUNOZ, N. & LINSELL, A. (1982). Epidemiology of primary liver cancer. In: *Epidemiology of Cancer of the Digestive Tract.* (Eds. Correa & Haenzel), The Hague: Martinus Nijhoff, p. 161.

NEWBERNE, P.M. & BUTLER, W.H. (1969). Acute and chronic effects of aflatoxin on the liver of domestic and laboratory animals – a review. *Cancer Res.,* **29,** 236.

OMATA, M., ASHCAVAI, M., LIEW, C.T. & PETERS, L. (1979). Hepatocellular carcinoma in the USA, etiologic considerations. *Gastroenterology,* **76,** 279.

PEERS, F.G. & LINSELL, C.A. (1973). Dietary aflatoxin and liver cancer. A population based study in Kenya. *Br. J. Cancer,* **27,** 473.

PEERS, F.G., GILMAN, G.A. & LINSELL, C.A. (1976). Dietary aflatoxins and human liver cancer: A study in Swaziland. *Int. J. Cancer,* **17,** 167.

PRATES, M.D. & TORRES, F.O. (1965). A cancer survey in Lourenco Marques, Portuguese East Africa. *JNCI,* **35,** 729.

PRINCE, A.M. (1978). Open discussion. In: *Viral Hepatitis.* (Eds. Vyas *et al.*), Franklin Institute Press, Philadelphia, p. 460.

PRZTBYLSKI, W. (1975). Formation of aflatoxin derivatives on TLC plates. *J. Ass. Off. Analyt. Chem.,* **58,** 163.

PURVES, L.R. (1973). Primary liver cancer in man as a possible short duration seasonal cancer. *S. Afr. J. Sci.,* **69,** 173.

REYS, L.L., PURCELL, R.H., HOLLAND, P.V. & ALTER, H.J. (1977). The relationship between hepatitis B virus infection and hepatic cell carcinoma in Mozambique. *Trop. Geogr. Med.,* **29,** 251.

ROSE, E.F. (1973). Esophageal cancer in the Transkei 1955–1969. *J. Natl Cancer Inst.,* **51,** 7.

ROSE, E.F. & FELLINGHAM, S.A. (1981). Cancer patterns in Transkei. *S. Afr. J. Sci.,* **77,** 555.

SHAFRITZ, D.A., SHOUVAL, D., SHEWMAN, H.I., HADZIYANNIS, S.J. & KEW, M.C. (1981). Integration of hepatitis B virus DNA into the genome of liver cells in chronic liver disease and hepatocellular carcinoma. *N. Engl. J. Med.,* **305,** 1067.

SHANK, R.C., BHAMARAPRAVATI, N., GORDON, J.E. & WOGAN, G.N. (1972). Dietary aflatoxins and human liver cancer. Incidence of primary liver cancer in two municipal populations in Thailand. *Fd Cosmet Toxicol.,* **10,** 171.

SKINHØJ. P. (1977). Hepatitis and hepatitis B antigen in Greenland. II: Occurrence and interrelation of hepatitis B associated surface, core and "e" antigen antibody systems in a highly endemic area. *Am. J. Epidemiol.,* **105,** 99.

SKINHØJ. P., HANSEN, J.P., NIELSONN, H. & MIKKELSEN, F. (1978). Occurrence of cirrhosis and primary liver cancer in an eskimo population hyperendemically infected with hepatitis B virus. *A, J. Epidemiol.,* **108,** 121.

SUNG, J.L., CHEN, D.S. & LIN, W.S. (1980). Hepatocellular carcinoma and hepatitis B virus. *Excerpta Med. Int. Congr. Ser.*, **502**, 631.

SZMUNESS, W. (1978). Hepatocellular carcinoma and the hepatitis B virus: Evidence for a causal association. *Prog. Med. Virol.*, **24**, 40.

TABOR, E., GERETY, R.J., VOGEL, C.L. *et al.* (1977). Hepatitis B virus infection and primary hepatocellular carcinoma. *J. Natl Cancer Inst.*, **58**, 1197.

TONG, M.J., SUN, S.C., BERTON, T. *et al.* (1971). Hepatitis associated antigen and hepatocellular carcinoma in Taiwan. *Ann. Intern. Med.*, **75**, 687.

TRICHOPOULOS, D., MACMAHON, B., SPARROS, L. & MERIKAS, G. (1980). Smoking and hepatitis B negative primary hepatocellular carcinoma. *J. Natl Cancer Inst.*, **65**, 111.

TRICHOPOULOS, D., PAPAERANGELOU, M., VIOLAKI, Ch., VISSOULIS, L., SPARROS, L. & MANOUSOS, O.N. (1976). Geographic correlation between mortality from primary hepatic carcinoma and prevalence of hepatitis to surface antigen in Greece, *Br. J. Cancer*, **34**, 83.

TRICHOPOULOS, D., GERETY, R.J. & SPARROS, L. (1978). Hepatitis and primary hepatocellular carcinoma in a European population. *Lancet*, **ii**, 1217.

TUYNS, A.J., LOUBIERE, R. & DUVERNET-BATTESTI, Fr. (1971). Regional variations in primary liver cancer in Ivory Coast. *J. Natl Cancer Inst.*, **47**, 131.

VAN DER HEEVER, A., PRETORIUS, F.J., FALKSON, G. & SIMSON, I.W. (1978). Hepatitis B surface antigen and primary liver cancer. *S. Afr. Med. J.*, **54**, 359.

VAN RENSBURG, S.J. (1977). Role of epidermiology in the elucidation of mycotoxin health risks. In: *Mycotoxins in Human and Animal Health.* (Eds. Rodericks *et al.*), Park Forest South: Pathotox Publ., p. 699.

VAN RENSBURG, S.J., VAN DER WATT, J.J., PURCHASE, I.F.H., COUTINHO, L.P. & MARKHAM, R. (1974). Primary liver cancer rate and aflatoxin intake in a high cancer area. *S. Afr. Med. J.*, **48**, 2508a.

VAN RENSBURG, S.J., KIRSIPUU, A., COUTINHO, L.P. & VAN DER WATT, J.J. '1975). Circumstances associated with the contamination of food by aflatoxin in a high primary liver cancer area. *S. Afr. Med. J.*, **49**, 877.

YEH, F.S., YAN, R.C., MOR, C.C., LIU, Y.K. & YANG, K.C. (1982). Research on etiological factors of hepatocellular carcinoma in Guangxi, China. *Proceedings of the Thirteenth International Cancer Congress, Seattle 1982*; p. 340.

WATERHOUSE, J., MUIR, C., SHANMUGARATNAM, K. & POWELL, J. (eds.). (1982). *Cancer Incidence in Five Continents.* Vol. IV Lyon: IARC Sc. Publ. No. 42; p. 812.

WATERHOUSE, J., MUIR, C., CORREA, P. & POWELL, J. (eds.). (1976). *Cancer Incidence in Five Continents.* Vol. III Lyon: IARC Sci. Publ. No. 15; p. 584.

ZIEGLER, J.L., ADAMSON, R.H., BARKER, L.F., FRAUMENI, J.F., GERIN, J. & PURCELL, R.H. (1977). National Institutes of Health International Workshop on hepatitis B and liver cancer. *Cancer Res.*, **37**, 4672.